# Experiences of nurse managers and practitioners on implementation of an evidence-based practice intervention

**DOI:** 10.4102/hsag.v27i0.1597

**Published:** 2022-02-28

**Authors:** Catherine Haulesi Chiwaula, Diana L. Jere

**Affiliations:** 1 Kamuzu College of Nursing, University of Malawi, Lilongwe, Malawi

**Keywords:** evidence-based practice, qualitative approach, qualitative design, facilitators of EBP, barriers of EBP, EBP experiences

## Abstract

**Background:**

An evidence based practice (EBP) research project was undertaken to implement EBP interventions utilising the Iowa model in order to build the capacity of the nurses in using research evidence to improve decision making and quality care.

**Aim:**

Exploring and understanding the experiences of nurse managers and practitioners who participated in the EBP change project.

**Setting:**

The study was conducted in the intensive care unit (ICU) of a tertiary hospital in Lilongwe district in Malawi.

**Methods:**

A qualitative approach and an exploratory-descriptive design was employed. The ICU was purposively selected as a unit where the EBP change project was implemented. A purposive sample of 10 nurse managers and practitioners was selected. Semi-structured interviews were conducted. All interviews were audio-recorded with a digital recorder and transcribed verbatim. Thematic analysis was applied to the transcripts.

**Results:**

The participants’ experiences of implementing EBP interventions were underpinned by four themes namely, evidence-based patient management, effective nursing care, competence in delivering EBP, and factors interplaying in EBP. Use of model, protocol and availability of supportive managers and team were major determinants of EBP.

**Conclusion:**

It is recommended to continue utilising the Iowa Model to facilitate building the EBP capacities of providers during scale up.

**Contribution:**

Utilising the Iowa Model facilitates building of the capacity and empowers frontline nurses to effectively develop, implement and evaluate discipline specific EBP changes needed to improve practice and optimum care.

## Introduction

Evidence Based Practice (EBP) is an approach for improving quality care (Stetler et al. 2007). It is when expert nurses use current research evidence in consideration of patients’ preferences to improve nursing care decisions, quality care, and patient outcomes (Melnyk et al. 2014). It is beneficial to patients, nurses, and the healthcare system. It results in provision of the most effective patient-centred care, increases professional growth and lead to the delivery of improved, safe and quality healthcare that enhances health outcomes, decreases disparity, and reduces costs (Hoffmann, Bennett & Del Mar 2013; Jylha et al. 2017; McGinty & Anderson 2008; Melnyk et al. 2012; Melnyk & Fineout-Overholt 2015).

Delivery of EBP has many challenges, ‘nurses relies heavily on personal experience and communication with colleagues rather than formal sources of knowledge’ (Gerrish et al. 2008:62). Hence, an EBP research project was conducted in the intensive care unit (ICU) utilising the Iowa model with the aim of implementing EBP interventions to improve the delivery of quality nursing care. The Iowa model comprises five action steps namely, (1) problem/triggers identification; (2) selection of an evidence-based intervention; (3) piloting the identified evidence-based intervention; (4) determining the appropriateness of adopting the practice beyond the pilot; and (5) scaling up the EBP change to other units/wards upon observation of positive outcomes (Buckwalter et al. 2017).

An extensive body of literature has shown that implementation of EBP is very challenging in nursing care practice with lower rates in Africa where Malawi is located. For example, the use of research evidence in nursing practice was at less than 50% in some areas of the European region (Adib-Hajbaghery 2009; Boström et al. 2009; Dalheim et al. 2012; Fairbrother et al. 2016; Melnyk et al. 2012; Melnyk & Fineout-Overholt 2015; Stokke et al. 2013; Warren et al. 2016; Weng et al. 2013). In the African region, the few studies that have been conducted have shown that there is extremely low application and use of research knowledge, with less than 35% in nursing practice (Agbedia et al. 2014; Mndzebele & Tshivhase, 2016; Mutisya 2015; Oluwatosin 2014; Owusu-Addo, Cross & Sarfo-Mensah 2017; Woldeyes 2012). In Malawi, the implementation of EBP among nurses is extremely low (Ministry of Health and Population 2012, 2015; Mulenga & Naidoo 2017; World Health Organization 2010). In order to improve the delivery of EBP in Malawi, the Iowa model was used to guide the nurses to implement evidence-based interventions and improve quality care provided to their patients.

### Problem statement

The implementation of EBP in Malawi is very low (Ministry of Health and population 2015; Mulenga & Naidoo 2017; WHO 2010). As such, a research project was undertaken using the Iowa model of EBP to guide the process of integrating the current research evidence with practitioner expertise and patients’ needs when making nursing care decisions thus improving the delivery of quality care. It was hypothesised that by following the steps of the Iowa model, nurses’ capabilities would be enhanced in identifying practices that do not work; asking a clinical question; accessing, selecting, introducing, testing and evaluating evidence-based fever interventions if they were effective as described in literature (Buckwalter et al. 2017). But their experiences were not known and therefore this study was conducted to explore their experiences. Experiences entailed the knowledge, skills, competences, emotional state, opinions, and beliefs gained through participating in the EBP research project (Merriam Webster Dictionary 1828).

### Study purpose

To explore and describe the experiences of nurse managers and practitioners who were involved in implementing EBP interventions in the ICU. Thus, the study serves as a guide in the upscaling of the interventions.

## Methodology

### Design

The study was informed by constructivist ontology which states that reality is multiple, subjective and mentally constructed (Creswell 2014; Rehman & Alharthi 2016). It was informed by interpretivist’s epistemology which states that knowledge of reality is subjective and that it is a social construction where people cannot be separated from their knowledge (Business Research Methodology 2018; Henn, Weinstein & Foard 2009). The researcher assumed that nurses developed thoughts, interpretations and the meaning of EBP after having hands on experience during implementation of the EBP change project. Thus, the nature of EBP experiences needed to be explored and interpreted through interacting, observing the reactions and describing the nurses’ experiences. In order to get the actual EBP realities, the researcher needed to ask questions, and understand and interpret the meaning of nurses’s EBP behaviours and voices through collecting in-depth data. As such, a qualitative approach and explorative- descriptive research design was employed.

To ensure that personal characteristics do not influence the research process the researcher maintained a reflective stance by bracketing, putting aside personal biases, assumptions, beliefs and past experiences (Tufford & Newman 2012). The researcher suspended or held in abeyance the interests, personal experiences, assumptions, feelings, and intuitions that could have influenced how the researcher viewed the study’s data. This made the researcher to be conscious of and mitigate the potential effects of preconception experiences.

### Setting

The study was conducted in the ICU of a tertiary hospital in Lilongwe district in Malawi. The target population consisted of 22 nurse managers and practitioners working in the ICU during the period when the EBP change study was conducted.

### Sampling method

Non-probability purposive sampling technique was employed to select 10 participants (until saturation was reached) who participated in implementing evidence-based fever intervention using the Iowa model. They were requested to volunteer to participate and share their experiences and lessons learnt.

### Data collection and tools

The researcher collected data using a semi-structured interview guide containing open-ended questions to collect in-depth information about the nurses’ EBP experiences. The interviews were recorded using a digital voice recorder. All participants granted permission to the researcher to record the interviews. The guide was pre-tested to determine its feasibility of collecting the desired information and was corrected before the main study.

### Data analysis

All interviews were audio-recorded and transcribed verbatim. The researcher analysed the data and transferred the texts that were produced into NVivo 12.0, which helped to organise, classify, sort, and arrange the information and examine relationships in the textual data (Edhlund & McDougall 2019; Zamawe 2015). In addition, co-coding was carried out by the co-researchers to identify common codes across the transcripts. Some members of the research team read data transcripts (e.g. field notes, interviews, reflection notes) and code them into generative categories manually. Consensus was reached before transferring into NVivo 12.0. Afterwards, a six-phase thematic analysis approach was used which included the researcher: familiarising self with the data, generating initial codes, searching for themes, reviewing potential themes, defining and naming the themes and producing the report (Braun & Clarke 2012).

### Trustworthiness

Trustworthiness refers to the degree of confidence in the data, interpretation, and methods used to ensure the quality of a study (Polit & Beck 2014). To ensure the trustworthiness of this study, credibility, transferability, dependability, and confirmability was observed. Credibility was achieved through member checking (Brown & Schmidt 2012). During member checking, the researcher returned the results to the participants to check for accuracy and resonance with their experiences. Credibility was also achieved by the researcher providing a detailed description of the processes followed in the study and their methods. Transferability was ensured by clearly describing the context of the study and checking the representativeness of the data. Representativeness was achieved through purposely selecting a mixture of participants who gave rich encompassing data to enable detection of the saturation levels. Thus, principal, senior and nursing officers were selected as participants in the interviews. Dependability was achieved by ensuring transparent and detailed description of the processes so that other reviewers can validate the results. Confirmabilit**y** refers to the objectivity of research during data collection and data analysis (Creswell 2014). Confirmability was done through accurate and detailed documentation of the methods and procedures, themes and sub-themes developed.

### Ethical considerations

The College of Medicine Research Ethics Committee (COMREC) of the University of Malawi approved the research (reference number: P.12/18/2549). It was ensured that our research was in accordance with the Code of Ethics for conducting research with human subjects. The hospital authorities and all the participants consented in writing. The participants were also guaranteed anonymity and confidentiality.

## Results

Ten participants were interviewed after reaching saturation. The participants provided rich insights into nurses’ experiences after piloting evidence-based fever interventions following the Iowa model. A total of four themes emerged from the data as follows: evidence-based patient care management, provision of effective nursing care, factors interplaying in EBP implementation, and competence in delivering EBP.

### Theme 1: Evidence-based management of patient

The results revealed that the participants had positive attitude towards implementing evidence-based fever interventions in ICU. They perceived that it led to evidence-based patient care management. Generally, the participants stated that they felt good about implementing EBP. Some warmly welcomed it, and some others said that it was a good development, innovation and initiative as illustrated in what they said. One participant felt that implementing EBP is a good innovation because it helped them to implement real evidence-based nursing care as shown in this comment:

‘EBP is a good innovation, because as nurses most of the times on fever management we use medication before doing other nursing care intervention. It has helped us to know our real nursing care intervention, evidence-based care, that’s good.’ (Participant 7, female, 36 years old, registered nurse midwife [RNM])

Another participant testified that it was a good initiative because implementation of the evidence-based intervention resulted in the improvement of nursing care delivery as demonstrated below:

‘I feel that It is a good initiative because previously before introduction of the intervention were just working haphazardly, but now, I think we are able to intervene accordingly, to follow evidence.’ (Participant 10, female, 45 years old, RNM)

These results have shown that evidence-based patient care management resulted from implementing approved best practices. The nurses in the ICU felt that the EBP study gave them the ability to effectively implement interventions that worked as best practices. This helped them to avoid haphazard care and provide standardised quality care for patients with fever. This is reflected in extracts below:

‘[… *A*]ccording to my observation, it has changed the way we manage fever and we used best practices. I have seen that there is a big improvement in quality … we provided standardized care.’ (Participant 6, male, 28 years old, nurse midwifery technician [NMT])

### Theme 2: Effective nursing care practices

#### Sub-theme 1: Improved practice

The nurses in the ICU observed that using the fever protocol that was developed at step three of the Iowa model led to effective nursing care practices. By following the fever protocol the nurses easily made decisions during care provision and improved their practice. This served as a clinical decision support at the point of care, so that no opportunities were missed to achieve fever control because it aided the decision-making process and it was their backing. In addition, it decreased the ambiguity of care provision, prevented guess work and thereby improved the nursing practice. This is illustrated in the expression below:

‘With the protocol, I can say, it was straightforward, helped to make decisions … because you knew the approach … you were organized and not stressed at all.’ (Participant 2, male, 26 years old, NMT)

#### Sub-theme 2: Evidence-based intervention

The nurses also recognised that effective nursing care practices resulted from implementing EBP interventions. Taking part in implementing an EBP intervention helped them to carry out an already proven intervention and enabled them to avoid errors and perform the correct measures that ensured patient safety and provision of quality care as illustrated in the quote below:

‘[… *D*]uring the change process we were using measures that already worked, some measures that were already implemented in some hospitals worldwide … they were already done and proven with evidence. It was safe and no errors.’ (Participant 3, female, 40 years old, senior nurse midwifery technician [SNMT])

### Theme 3: Factors interplaying in evidence-based practice implementation

The perspectives of the participants in this study has revealed that achieving evidence-based practice using Iowa model is an interplay of a number of factors. These were seen as drivers, impediments and elements enhancing EBPs. These are described in the following sections.

#### Sub-theme 1: Drivers of evidence-based practice implementation

The participants were of the view that the drivers of effective EBP innovation which was implemented in the ICU were as follows: availability of local resources, implementing nursing specific EBP intervention, team work, knowledgeable providers, use of protocol, ownership of research and incentives. The participants said that the availability of local resources facilitated smooth implementation of the evidence-based innovation and aided in good performance as reflected in the statement below.

‘The resources, were locally available. … So, knowing that the resources are there I think it affected positively to the implementation.’ (Participant 1, female, 33 years old, RNM)

The participants felt that the study was successful because it was a nursing-specific research. Through participation in the EBP study, the nurses were exposed to evidence-based nursing practices. They realised that it was critical to the nursing provision and encouraged them to implement a nursing intervention that promoted the provision of optimal nursing care. An example of such view is evident in the following excerpt:

‘[… *Y*]ou brought things …, which we normally do all time as nurses, …, it was good to improve our own care as nurses, ….’ (Participant 2, male, 26 years old, NMT)

The participants appreciated the approach to EBP using the Iowa model because it encourages team work. They mentioned that they worked as a team during all stages of implementing the EBP study. They felt that the study was a success because they collaboratively worked together to introduce and pilot an evidence-based change intervention in the ICU as illustrated in the following quotes:

‘It was successful because of team coordination, …. I could say that it was the teamwork which enabled us to work together during the patient management steps.’ (Participant 5, male, 28 years old, RNM)

The participants perceived that the EBP implementation was successful because they were knowledgeable. They felt that they acquired the knowledge and skills necessary to implement an evidence-based innovation. These influenced their performance positively and they acted as facilitators of good performance. This is expressed in the statement below:

‘I think basically knowledge facilitated, because after the nurses were given knowledge and competences on management of fever …, we took it positively, we started utilizing it, and we succeeded.’ (Participant 10, female, 45 years old, RNM)

The participants realised that the protocol was a tool that contained a standard set of nursing actions and it guided the delivery of care to patients with fever. They also said that availability of the protocol made the study successful. Participant number 09 provided a good description of this perspective:

‘[… *T*]o me it is the way the protocol was designed it helped very much, it was easy to follow … it made our work easy, it guided us.’ (Participant 9, female, 32 years old, RNM)

The participants reiterated that the ownership of research by participants gave them the power to control the study because they took it as their own. Involvement of the providers at all stages made them perceive that their presence in the clinical area was important in achieving objectives of the study. Some of the participants expressed the following statements:

‘There was good engagement … as a result, we took it as if it’s our own study, ownership was there, so I think that’s why people accepted it and took it positively and run with the research vision.’ (Participant 10, female, 45 years old, RNM)

The participants also perceived that good performance was influenced by the allowances which were given to them at the end of the month as an incentive. They believed that allowances were a way of rewarding the great work which they were doing. This motivated and inspired the nurses to work and achieve the desired goals of care. This is illustrated in the quotes below:

‘I can also say allowances we were receiving at the end of the month, … encouraged us to work. It made us to be eager and work very hard, but also in the right way, to be productive and care for fever cases.’ (Participant 4, female, 48 years old, SNMT)

#### Sub-theme 2: Impediments of evidence-based practice implementation

The study revealed that the nurses encountered some impediments during implementation of the evidence-based innovation in the ICU. The problems included shortage of staff, increased workload, incomplete documentation, fear of initiating an evidence-based innovation, negative attitude to change, and lack of proper handovers by some participants.

The participants said that sometimes there was shortage of nurses on duty which in turn increased the workload. In the ICU, the required nurse patient ratio is 1:1 and they were expected to do observations hourly as required. On some days, the ratio was one nurse to two patients. This made it difficult in observing hourly monitoring time. In such cases, they prioritised patients, and the patients with abnormal temperature were in the category of priority patients. This is reflected in what one participant said:

‘[… *B*]ut sometimes there was shortage of staff which would have affected the care given to patients, although not, but we prioritized the care of patients with fever.’ (Participant 7, female, 36 years old, RNM)

The results have shown that incomplete documentation was one of the challenges at first as demonstrated in the quote below.

‘Sometimes if your friends have not documented well, when coming in, it was difficult to know, you were like where can I start from, but later after the meeting everyone was documenting.’ (Participant 3, female, 40 years old, SNMT)

The study shows that some nurses had fear of implementing the intervention that they have never tried before, such as use of ice packs and cool intravascular fluids in the management of fever. In this case, they had to observe others before trying it as illustrated in the quotes below:

‘[… *W*]hat was difficult was, ice packs and intravascular cooling because I have never used it before …, I had fear, … But I had to use icepacks after observing others and things worked.’ (Participant 10, female, 45 years old, RNM)

The participants also observed negative attitudes such as lack of interest in some of their fellow nurses. As a result, they were not serious when on duty. Therefore, working in a team helped to provide collaborative care and the persuasion from other team members enabled them to revisit their behaviours that seemed less effective and thus they were brought on board as indicated in the following statement:

‘Challenges, yes were still there … as ICU we work as a team, some people you know when change is being implemented, some people do not take it positively. But otherwise most of the nurses took it positively, but few were not serious … But in the end seeing and working with friend they came along and provided quality care.’ (Participant 10, female, 45 years old, RNM)

#### Sub-theme 3: Elements enhancing evidence-based practice implementation

The participants were given a chance to assess their performance in relation to that of the facilitators and challenges experienced and come up with what they felt could enhance the implementation of EBP during scale up. They perceived that for them to work better in the next phase, there was a need to focus on the following: continuing the use of fever protocol, ensuring regular training, improving documentation, and combining fever control methods.

The participants in this study experienced that using the fever protocol could help to maintain provision of the desired care to patients with fever. Some nurses think that the protocol helped ensure that patients with fever get the right care because it guided on what steps to take to provide the expected care and treatment as pointed out in the quote below:

‘When we have done all, tepid sponging, ice pack, antipyretics, and reaching intravascular cooling the fever was resolved totally … I think if we can do step by step as we were doing following the protocol, it can help to improve care.’ (Participant 2, male, 26 years old, NMT)

The participants realised that providing regular training opportunities to the nurses is a promising strategy for promoting the delivery of EBP. It empowers nurses to be EBP providers and ensures excellence in clinical decision making. This is reflected in the quotes below:

‘In order to improve delivery of our intervention, I think there is need for regular …. Reorientation and training to give us knowledge.’ (Participant 9, female, 32 years old, RNM)

Another participant said the following:

‘As I said before, training is paramount, it provides knowledge, when nurses are given knowledge on management of fever, it is easier to succeed.’ (Participant 10, female, 45 years old, RNM)

The participants observed that improving documentation was also an essential element of safety and quality. Documentation of fever management need to be integrated into patients’ files to communicate with other members of the healthcare team. They felt that there was need to use the Subjective Objective Assessment Plan Implementation and Evaluation (SOAPIE) format to document care of patients with fever. One participant said the following:

‘In terms of improvement, … documentation of the care that is provided to the patient … we need to integrate in patients’ files …, I think we need to improve and use our SOAPIE.’ (Participant 10, female, 45 years old, RNM)

Some participants felt that combining other methods of fever control could be more effective than using a single method alone as shown in the quote below. However, there is need to conduct further studies to find out which methods can be combined and if combining methods of fever reduction in Malawi will be safe or not.

‘I think combining method is important, I think exposure and tepid responding … can go hand in hand. We shouldn’t wait, exposing one hour then starting tepid sponging. I think exposure and tepid sponging can work hand in hand because it is the same mode of evaporation … I think that we can be doing simultaneously and could be effective.’ (Participant 9, female, 32 years old, RNM)

### Theme 4: Competence in delivering evidence based practice

#### Sub-theme 1: Capability to deliver evidence based practice

Generally, nurses valued their participation in this research. They perceived that following the Iowa model helped them to develop the capability and capacity to deliver evidence-based care. Capability is the ability to perform or achieve certain actions or outcomes through acquiring the required knowledge, skills and level of capacity (Collins Dictionary 2020). The nurses who participated in the study developed a set of knowledge, skills, behaviour and disposition through providing EBP. Following the steps of the Iowa model made nurses capable of providing EBP, because they also gained experience, confidence, will, and power to improve their expertise through taking action. This is illustrated in the expression below:

‘We have gained knowledge, experience … unlike sometimes we like using short cuts, but now we have seen and experienced how we can do perfect things ….’ (Participant 2, male, 26 years old, NMT)

Another participant perceived that they were capable because they acquired the necessary skills to implement EBP. This is demonstrated in the quote below:

‘… I have added some skills, some knowledge actually, especially with this intravascular cooling. I used to think that it is dangerous to give cold fluids, but this has enlightened me. That it is possible to use fluids cooled up to 4 degrees and so I have gained the knowledge and skills.’ (Participant 1, female, 33 years old, RNM)

The results also revealed that their capability arose from the confidence they gained through participating in taking action using the Iowa model as indicated in the below quote:

‘Using the Iowa model, helped you to gain confidence, when implementing your care, because you knew it was approved that it works. Then … you had confidence that it will work ….’ (Participant 3, female, 40 years old, SNMT)

#### Sub-theme 2: Capacity to deliver evidence based practice

Capacity means the ability to do something, in our case to provide EBP (Collins Dictionary 2020). The nurses perceived that they developed the capacity to provide the best care backed by evidence, EBP innovation, and the Iowa model including participating in a team of EBP providers. They understood the Iowa model as a guiding tool, backup and reference tool that provided them with the direction on how to implement an EBP action. They also believed that the model contains approved action steps that helped them to develop the capacity to perform effectively. The participants views are shown in the quote below.

‘[…*U*]sing the Iowa model you were able to do EBP, … it’s like you have the capacity, a backup and guidance that you are using something that was used and worked … you have that confidence and guidance to do it ….’ (Participant 3, female, 40 years old, SNMT)

The participants were of the opinion that the Iowa model was a planning tool that helped to improve nursing care management as illustrated in the quote below:

‘It reminded us of our nursing, like planning, how to do our nursing care plan, it was taking us somewhere apart from the daily routine in the ward, were practicing the exact practices of fever management. It was guiding us ….’ (Participant 4, female, 48 years old, SNMT)

## Discussion

The study explored and described the experiences of nurse managers and practitioners that participated in introducing EBP fever intervention in nursing care delivery using the Iowa model. The findings revealed that during execution of the EBP study, most nurses adopted a positive stance towards EBP implementation which empowered them to take up their EBP role. These results are similar to a number of studies which revealed that nurses had positive attitudes towards EBP as a driver of EBP (Al-Maskari & Patterson 2018; Elarab et al. 2012; Linton & Prasun 2013; Williamson et al. 2015). In this case, positive attitudes act as a catalyst for scale up and sustainability.

The participants believed that their transformation from non EBP providers to EBP providers was made possible because of the knowledge and skills acquired through taking action. Their decision-making capacity developed to facilitate the provision of optimum nursing care. This concurs with the opinion of Jylha et al. (2017) who stated that implementing EBP results in enhancing the decision-making capacity of practitioners to deliver effective, efficient, and good quality healthcare and improved healthcare outcomes.

In addition, the nurses perceived that availability of local resources, implementing nursing-specific EBP intervention, team work, use of protocol, and ownership of research were the contributing factors to EBP implementation. The participants also felt that combining fever control methods, adjusting monitoring time frame, ensuring regular training, and improving documentation could sustain the implementation of EBP efforts. Moreover, they testified that their confidence in making evidence-based decisions also improved. These results are supported by several authors who also found that the availability of resources, guidelines, and ownership are facilitators of EBP (Brown et al. 2009; Hoyiso, Arega & Markos 2018; Jylha et al. 2017; Kueny et al. 2015; Brown & Schmidt 2012). The results highlights that subsequent efforts to roll out EBP implementation are likely to succeed if we focus on utilising the fever protocol or developing a guideline by trying out combining fever control methods, adjusting monitoring time frame, ensuring regular training, and improving documentation.

Furthermore, the participants observed that factors like shortage of staff, increased workload, incomplete documentation, fear of initiating an evidence-based innovation, and negative attitude to change impeded implementation of EBP. These results correspond with the result of a systematic review of 11 studies which demonstrated among others facts, that inadequate resources, inadequate EBP skills and knowledge of practitioners and negative attitudes of practitioners act as barriers to EBP implementation (Gray et al. 2013). Discovering these impediments are early critical elements which needs to be resolved during the process of EBP implementation.

The most encouraging result was that after practising and experiencing implementation of evidence-based fever interventions using the Iowa model, it led to improved EBP knowledge, skills, confidence, and improved emotional states, which thus led to efficient and effective decision making and standardised EBP approach. This agrees with the study which states that the process of EBP involves using professional experience, skills and training, which leads to increased knowledge empowerment, improved skills, development of expert roles and enhanced clinical judgement (Jylha et al. 2017). It means the participants developed EBP clinical expertise for successful scale up of the interventions. Clinical expertise refers to the integration of accumulated knowledge, skills and information from education and clinical care experiences in making care decision (Burns & Grove 2010; McHugh & Lake 2010). Thus, they developed clinical expertise which influenced their clinical judgement. This led to effective and efficient critical decisions that helped them to choose the best options that eventually enabled them to provide quality healthcare. This is in accord with the studies that found that, if nursing practice is based on scientific research evidence, it helps nurses to select the best option from a range of choices. This results in a nursing practice being more likely to be safe, effective, cost-effective and producing the intended patient outcomes (International Nurses Day 2012; Stevens 2013; Titler 2008). This discovery is important for effective scaling up of the EBP interventions. To achieve this, the nurses need to be knowledgeable, have well-developed skills and be experienced.

## Conclusion

The findings of this qualitative study have described the experiences of nurses during EBP action. Overall, the participants felt that taking part in the EBP study allowed them to develop the capacity and capability to implement EBP. They also perceived that discovering the factors interplaying in EBP was essential to promote successful delivery of EBP. They believed that using the Iowa model was a catalyst to EBP. It directed on the formulation of specific strategies designed to change barriers into enablers during the process of EBP implementation. For successful scale up in evidence-based care abilities, it is recommended to focus on changing impediments like shortage of staff and increased workload, incomplete documentation, fear of initiating an evidence-based innovation, and negative attitude into enablers to enhance the EBP implementation. In addition, there is need to concentrate on the elements that can augment EBP action like developing protocols or guidelines and building a supportive team.

### Limitation

The study only focused on nurses who participated in the study. The patients who received care were not interviewed because of their condition. They were unconscious. The patients would have been interviewed to find out their experience as well.
